# Peripheral perfusion response to semiorthostatic stress: a simple method for assessing autonomic dysfunction in sepsis?

**DOI:** 10.62675/2965-2774.20240090-en

**Published:** 2024-11-14

**Authors:** Lilian Barth Guimarães, César Maistro Guimarães, Hipólito Carraro, Jamil Assreuy, Igor Alexandre Côrtes de Menezes

**Affiliations:** 1 Universidade Federal do Paraná Hospital de Clínicas Intensive Care Unit Curitiba PR Brazil Intensive Care Unit, Hospital de Clínicas, Universidade Federal do Paraná – Curitiba (PR), Brazil.; 2 Hospital e Maternidade São José dos Pinhais Intensive Care Unit São José dos Pinhais PR Brazil Intensive Care Unit, Hospital e Maternidade São José dos Pinhais - São José dos Pinhais, (PR), Brazil.; 3 Universidade Federal de Santa Catarina Department of Pharmacology and Center for Biological Sciences Florianópolis SC Brazil Department of Pharmacology and Center for Biological Sciences, Universidade Federal de Santa Catarina - Florianópolis (SC), Brazil.

**Keywords:** Autonomic nervous system, Stroke volume, Heart rate, Arterial pressure, Hemodynamics, Microcirculation, Perfusion index, Prognosis, Sepsis

## Abstract

**Objective:**

To evaluate whether the perfusion index response to semiorthostatic stress can be used to monitor the sympathetic-vascular response in sepsis patients.

**Methods:**

Three groups were studied: Group A (30 healthy patients), Group B (30 critically ill patients without sepsis), and Group C (92 septic patients). The patients underwent a semiorthostatic stress test (head elevation from 0 to 60 degrees), and hemodynamics, perfusion index values and cardiac ultrasound data were evaluated. SOFA scores were also evaluated in septic patients, comparing those with increased and decreased perfusion indices after the test.

**Results:**

After the test, Group A presented significant reductions in stroke volume (p < 0.01) and the cardiac index (p < 0.05), with increases in heart rate (p < 0.05) and mean arterial pressure (p < 0.001). These responses were not observed in Groups B and C. In the individual analysis of Group A, there was a decrease in the perfusion index (p < 0,001), whereas in Groups B and C, the response was heterogeneous. Additionally, septic patients who had a reduced perfusion index after the test had a significant decrease in the SOFA score at 72 hours compared with the group with an increased perfusion index (p < 0.05). However, the delta-SOFA score did not differ between the groups.

**Conclusion:**

The perfusion index response to semiorthostatic stress in sepsis patients is a simple and inexpensive method that can be used to detect the sympathetic-microvascular response at the bedside and appears to have prognostic value.

## INTRODUCTION

Sepsis is an issue that continues to cause high mortality.^(1)^ There is an ongoing need to develop new clinical and hemodynamic monitoring approaches to improve patient prognosis. Significant advances have been made, especially through bedside assessment of microvascular disorders via portable devices.^(2)^ When such devices are used, an unequivocal association between these disorders and unfavorable outcomes has been demonstrated, and these findings provide significant insights into the pathophysiology of sepsis. An association with poor prognosis was found regardless of whether the patients were evaluated early or late in relation to the duration of the hemodynamic management of sepsis.^(2)^

The autonomic nervous system (ANS), and specifically the sympathetic nervous system (SNS), is crucial for the regulation of perfusion. However, in cases of sepsis, there are disturbances in the modulation of the ANS, along with the likely harmful effects of exogenous sympathetic mediators (e.g., vasoactive drugs) that may contribute to an unfavorable prognosis.^(3)^ Extensive research has linked impaired SNS with the progression of circulatory dysfunctions, using direct measures (surgical or pharmacological interventions in animals) or indirect estimates of autonomic activity in patients/animal models.^(4)^ Furthermore, the ANS response can occur quickly to restore homeostasis. Clinical signs of ANS dysregulation may therefore be early warning signs of poor prognosis attributed to cardiovascular decline.^(4)^

In addition to its role in the microcirculation, the ANS is also of central importance in orthostatic homeostasis, involving circulatory and neuroendocrine responses to maintain adequate organ perfusion, especially in the upright posture.^(5)^

Modern oximeters calculate the perfusion index (PI), derived from photoplethysmographic signals. The perfusion index is considered a noninvasive measure of peripheral microcirculatory perfusion^(2,6)^ and has high sensitivity to changes in sympathetic stimulation^(6)^ and different body positions.^(7)^ Recently, the hemodynamic responses of the PI values to changes in body position have been used as a monitoring tool in fluid therapy, as seen in the passive leg raising test.^(8)^ However, the use of positional changes as a method to evaluate the ANS response has not been tested.

Considering the prognostic role of microcirculatory/perfusion disorders and autonomic dysfunction in sepsis, the role of the SNS in the circulatory response to positional changes, and the high sensitivity of the PI to positional changes, our hypothesis in the present study is that the PI response to semiorthostasis may constitute a simple and easy bedside test to monitor autonomic activity in sepsis. The objective of this study to evaluate whether the PI response to semiorthostatic stress can be used to monitor the sympathetic-vascular response in sepsis patients.

## METHODS

### Study design, setting and participants

The research was approved by the Human Research Ethics Committee of *Complexo do Hospital de Clínicas* of the *Universidade Federal do Paraná* (UFPR), CAAE: 38786320.9.0000.0096, protocol: 5.075.251), and all participants or legal guardians received and signed the informed consent in writing, consenting to participate in the study. This observational study was conducted in three intensive care units (ICUs) in Curitiba and São José dos Pinhais, Paraná, Brazil, between February 2020 and June 2023. During the study, all consecutive adult patients (≥ 18 years) admitted to the ICU or within 24 hours after sepsis onset among patients previously admitted for other causes were considered for enrollment.

The exclusion criteria were pregnancy, death within 72 hours, use of mechanical ventilation, severe obstructive arteriopathy, clinical contraindication to bed mobilization, and patient refusal to participate in the study.

According to the latest consensus, "sepsis" is identified by the presence of an infection associated with acute organ dysfunction, as indicated by an increase in the Sequential Organ Failure Assessment (SOFA) score of two points or more.^(9)^ Septic shock is a subset of sepsis in which, despite adequate fluid resuscitation, persistent hyperlactatemia (arterial lactate ≥ 2mmol/L) is associated with the need for vasopressors to achieve a mean arterial pressure (MAP) ≥ 65mmHg.^(9)^

### Study protocol

All patients were treated following a standard protocol adapted from the "Surviving Sepsis Campaign" (SSC) guidelines.^(1)^ Infectious sources, clinical examination, laboratory testing, culture collection, and image acquisition were performed. Broad-spectrum antibiotics were administered within the first hour based on the suspected source. Patients received fluid resuscitation with up to 30mL/kg of crystalloid fluid during the first 3 hours of diagnosis. Additionally, according to the criteria of the physician in charge and if there was an individual clinical indication, fluid resuscitation was maintained until there was a lack of reaction to the passive leg test or no respiratory variance in the inferior vena cava diameter. If the MAP remained less than 65mmHg, progressive doses of norepinephrine were used to normalize the MAP. Vasopressin was associated with norepinephrine in refractory cases.

Patients were assessed after fluid resuscitation between 6 and 24 hours after diagnosis. All patients were evaluated following at least one hour of hemodynamic stability (without any modification of vasoactive drug doses if in use). The information collected at enrollment included demographic characteristics, sepsis data, microbiology results, comorbid chronic illnesses, and severity scores such as the Acute Physiology Chronic Health Evaluation II (APACHE II) and SOFA scores. In addition, hemodynamics (MAP, heart rate [HR], urinary output), gasometric parameters (central venous oxygen saturation [ScvO_2_]_,_ venous to arterial carbon dioxide difference [Pv-aCO_2_]), biomarkers (procalcitonin, C reactive protein [CRP]), and lactate levels were obtained before the semiorthostatic test was performed.

**Figure 1:**
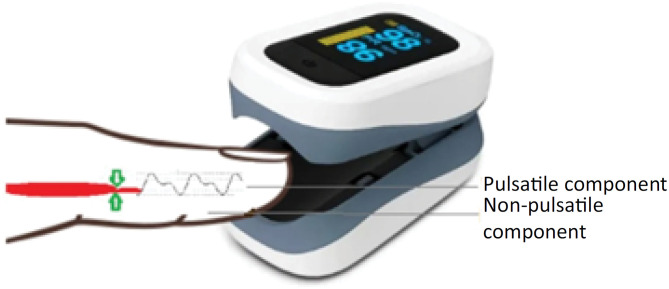
Peripheral perfusion indices and pulsatile and nonpulsatile components.

The perfusion index represents the relationship between pulsatile and nonpulsatile components of the photoelectric signal originating from pulse oximetry (Figure 1). The principle of oximetry is based on two light sources with different wavelengths (660nm and 940nm) emitted through the cutaneous vascular bed of a finger or earlobe. Using a two-wavelength system, the nonpulsatile component (such as connective tissue, bone, and venous blood) is then discarded, and the pulsatile component is used to calculate arterial oxygen saturation. As only the pulsatile component of the signal varies according to blood flow fluctuations (secondary to vasodilation or vasoconstriction), PI is considered a valid method for evaluating peripheral perfusion.^(10)^ The perfusion index was measured by placing an oximeter probe (MINDRAY, Shenzen, China) on the index finger.

The intensivists were blinded to the test values to avoid possible treatment bias. Finally, the patients were followed for 28 days after diagnosis or until discharge.

### Cardiac ultrasound

All transthoracic 2D echocardiography was performed by the same physician, who was blinded to the clinical data and fully trained in echocardiography. An ultrasound device from Philips (Affiniti 70G; Andover, MA, USA) was used. In this study, the collected parameters included the ejection fraction (EF), left ventricular outflow tract, stroke volume (SV) and velocity time integral (VTI). The cardiac index (CI) and cardiac output (CO) were calculated from the data.

### The semiorthostatic test

Patients were kept in the supine position, with the headboard at 0° for 5 minutes. PI, capillary refill time (CRT), and cardiac ultrasound data (SV, CI, CO, EF) were subsequently recorded. After this period, the headboard was elevated to 60° and maintained in that position for 3 minutes, and the same data were recorded. This experimental protocol was based on the standardized assessment of orthostatic hypotension in outpatients.^(11)^

The participants were subsequently divided into three groups:

- Group A - healthy control group: ICU workers without evidence of acute infections.
- Group B - critically ill patients without sepsis: patients admitted to the ICU for other illnesses without meeting sepsis diagnostic criterion.
- Group C - septic patients (< 24 hours): patients admitted to the ICU and diagnosed with sepsis.

Additionally, sepsis patients were further divided into two groups, namely, those with increased peripheral perfusion after the test (PI post test - PI pretest > zero arbitrary units) and patients with reduced peripheral perfusion after the test (PI post test - PI pretest ≤ zero arbitrary units).

### Outcomes

The primary outcome of this study was a comparison of the hemodynamic variables and PIs after semiorthostatic testing between Groups A, B and C.

The secondary outcome of this study was a comparison of organic dysfunctions in the groups in which the PI increased or decreased after the test, as measured by serial SOFA scores (24 and 72 hours) and the delta-SOFA score after 72 hours.

### Statistical analysis

To assess the normality of the sample, the Shapiro–Wilk test was used. Parametric data are presented as the mean ± standard deviation (SD), and nonparametric data are presented as the median and interquartile range. Proportions are represented as percentages. Comparisons between groups in terms of the delta-SOFA score and hemodynamics were performed via the Mann–Whitney test. For the serial evaluation of the SOFA score (24 and 72 hours), the Mann–Whitney test (intergroup) and Wilcoxon signed-rank test (intragroup) were used with correction for multiple comparisons via the Holm–Bonferroni method. To calculate the sample size, we used data from a pilot study with the first including 30 septic patients (which was used for comparison with Groups B and C). Thus, patients who had a reduced PI constituted two-thirds of the sample, and this group had an average delta-SOFA score (72 hours) of zero points, with a standard deviation of approximately 3 points. Additionally, in this study, a minimum difference of 2 points between the groups was necessary for clinical relevance in relation to the delta-SOFA score.^(12)^ Because the data are nonparametric, the sample was also inflated by 15%.^(13)^ Considering all these aspects, we estimated a total sample of 92 patients with sepsis (Group C).

For the clinical-hemodynamic analysis, the sample sizes of Groups A and B had were calculated based on previous studies in the literature.^(14,15)^ As a result, we estimated 30 patients in each group to verify an approximately 25% decrease in SV after the test. The sample power chosen was 80%, with an alpha error of 5%.

This study followed the STROBE guidelines for reporting results.

## RESULTS

**Figure 2:**
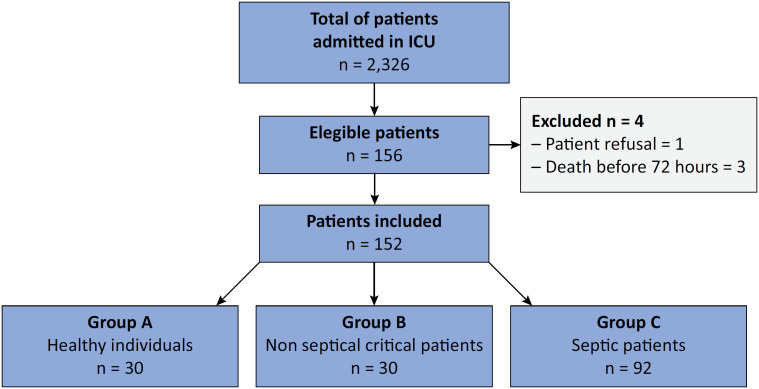
Study patients.

A total of 152 patients were included in study of the semiorthostatic test, as shown in figure 2. Importantly, a large part of this study was carried out during the coronavirus disease 2019 (COVID-19) pandemic, and owing to internal limitations in service due to known medical care difficulties during this period, the feasibility of data collection was frequently interrupted, delaying the completion of the study. The clinical, demographic and hemodynamic data are presented in table 1. These data characteristically describe a heterogeneous and critically ill population. Critically ill patients had a high prevalence of hypertension and diabetes in addition to comorbidities common in other intensive care cohorts (such as cancer and chronic lung disease). Additionally, the selected patients included surgical and nonsurgical patients (in both the septic and nonseptic groups).

ICU - intensive care unit.

**Table 1:** Demographic, clinical and hemodynamic data in healthy controls (Group A), nonseptic critically ill (Group B) and septic patients (Group C)

Parameters			Group A n = 30	Group B n = 30	Group C n = 92	Group C Survivors n = 67	Group C Non survivors n = 25	p value
Clinical data								
	Age (years)		37.3 (11)	62,3 (16.17)	55,2 (16.6)	52.9 (16.6)	61.9 (11.45)	0.007*
	Gender							
		Male	18 (60)	11 (36.6)	60 (65.2)	42 (62.6)	18 (72)	0.46
		Female	12 (40)	19 (66.3)	32 (34.8)	25 (37.3)	7 (28)	
Comorbidities								
	Diabetes Melitus		0	10 (33.3)	23 (25)	17 (25.4)	6 (24)	> 0.99
	Hypertension		0	16 (53.3)	38 (41)	27 (40.3)	11 (44)	0.81
	Chronic kidney disease		0	3 (10)	7 (7.6)	3 (4.5)	4 (16)	0.84
	Cardíac failure		0	9 (30)	17 (18.5)	14 (20.9)	3 (12)	0.38
	Hepatic failure		0	1(3,3)	7 (7.6)	4 (6)	3 (12)	0.38
	Cerebrovascular disease		0	3 (10)	4 (4.3)	3 (4.5)	1 (4)	> 0.99
	Chronic lung disease		0	5 (16.6)	15 (16.3)	11 (16.4)	4 (16)	> 0.99
	Cancer		0	0 (0)	22 (23.9)	13 (19.4)	9 (36)	0.10
	Immunosuppressed		0	1 (3.3)	15 (16.3)	12 (17.9)	3 (12)	0.75
Source of infection								
	Pulmonary		N/A	-	45 (48.9)	31 (46.3)	14 (56)	0.48
	Abdominal		N/A	-	22 (24)	15 (22.4)	7 (28)	0.59
	Urinary		N/A	-	13 (14.1)	13 (19.4)	0 (0)	0.01*
	Others		N/A	-	12 (13)	8 (11.9)	4 (16)	0.79
	Positive culture		N/A	-	72 (78)	52 (77.6)	20 (80)	> 0.99
	Positive hemoculture		N/A	-	13 (14.1)	14 (20.9)	5 (20)	> 0.99
	COVID-19 confirmed		N/A	-	32 (34.8)	21 (31.3)	11 (44)	0.32
Scores and biomarkers								
	SOFA - 24 hours		N/A	2.2 (1.9)	5.9 (2.7)	5.5 (2.5)	6.7 (3.1)	> 0.99
	SOFA - 72 hours		N/A	1.6 (1.6)	5.8 (3.9)	4.5 (3.0)	8.9 (4)	0.0001†
	APACHE II		N/A	10.7 (5.6)	13.7 (5.6)	12.8 (5.2)	16 (5.9)	0.004‡
	C-reactive protein (mg/dL)		N/A	4.4 (6.8)	17.2 (11.2)	17.2 (11.2)	17.6 (11.2)	0.71
	Procalcitonin (ng/mL)		N/A	-	12/0.2 (0.1 - 0.3)	10/0.15 (0.08 - 0.3)	3/0.3 (0.2 - 3.3)	0.001‡
Postresuscitation hemodynamics								
	Mean arterial pressure (mmHg)		89 (82 - 93.5)	85 (80.2 - 93)	85 (76 - 92)	85 (77 - 94)	84 (74 - 90)	0.29
	Heart rate (bpm)		70.6 (11.8)	76 (12)	94 (22)	95 (20)	93 (26)	0.42
	Capillary refill time (s)		2 (2 - 2)	2 (2 - 3)	3 (2 - 3)	2 (2 - 3)	3 (2 - 3)	0.28
	Perfusion index (a.u.)		5.3 (2.8 - 7.4)	3.7 (1.2 - 6.8)	3.2 (1.4 - 6.0)	3.2 (1.4 - 5.9)	3.1 (0.9 - 6.2)	0.33
	Velocity time integral (cm)		22.5 (2 - 24)	25.5 (19 - 27.2)	19.8 (17 - 23.5)§	19.3 (17.7 - 22)	22 (18.7 - 28.6)	0.41
	Cardiac index (L/minute/m²)		2.8 (2.2 - 3.1)	3.1 (2.4 - 3.5)	3.2 (2.8 - 3.9)§	3.1 (2.2 - 3.5)	3.7 (3.3 - 4.2)	0.021*
	Ejection fraction		66 (65 - 68)	55 (46.5 - 61.5)	61 (56.8 - 65)§	63.6 (58 - 67)	56.5 (55 - 62.2)	0.02*
	Cardiac output (L/minute)		5.1 (4 - 5.9)	5 (4.5 - 6.6)	6.2 (5.1 - 7.1)§	5.7 (4.6 - 7)	6.7 (6 - 7.5)	0,14
	Stroke volume (mL)		72,5 (62 - 80.7)	75 (59 - 86)	74 (61 - 90.5)§	73 (58.7 - 87)	80 (71 - 102)	0.25
	Percentage shortening (%)		36 (35 - 38)	30 (23.2 - 32)	33 (30 - 35)§	34 (32 - 36)	29.6 (29 - 31.5)	0.03*
	Left ventricular outflow tract (cm)		2.0 (1.9 - 2.1)	2 (1.9 - 2.1)	2.1 (2.1 - 2.2)§	2.1 (2.1 - 2.12)	2.2 (2.1 - 2.2)	0.09
	Vasoactive drugs use		N/A	0 (0)	28 (30.4)	20 (29.8)	8 (32)	> 0.99
	Norepinephrine dose (µg/kg/minute)		N/A	0 (0)	0.3 (0.1 - 0.65)	0.25 (0.1 - 0.6)	0.5 (0.2 - 0.9)	0.69
	Vasopressin use		N/A	1 (1.63)	4 (4.3)	1 (1.5)	3 (12)	0.05
	Arterial lactate (mmol/L)		N/A	18/1.2 (0.8 - 1.7)	71/1.8 (1.5 - 2.7)	50/1.8 (1.5 - 2.6)	21/1.6 (1.3 - 2.9)	0.62
	Urinary output (mL/kg/hour)		N/A	26/0.7 (0.7 - 0.8)	62/0.6 (0.5 - 0.8)	48/0.65 (0.49 - 0.8)	14/0.5 (0.5 - 0.7)	0.09

SOFA - Sequential Organ Failure Assessment; APACHE II - Acute Physiology Chronic Health Evaluation II.

* = p < 0.05;

† = p < 0.001;

‡ = p < 0.01;

§ Data were calculated for the first 32 septic patients. p value: Group C survivors *versus* nonsurvivors. The results are expressed as the means (standard deviations). n (%), n/median (interquartile range) or median (interquartile range).

As shown in table 2, the semiorthostatic stress test caused a reduction in SV and CI in the healthy control group (Group A), with compensatory increases in HR and MAP. Concomitant with these macrohemodynamic responses, there was a significant decrease in peripheral perfusion measured by the PI. This integrated response was lost in the nonseptic (Group B) and septic (Group C) critically ill patients.

**Table 2:** Macrohemodynamics and peripheral perfusion in healthy controls (Group A), nonseptic critically ill patients (Group B) and septic patients (Group C) before (0°) and after the semiorthostatic stress test (60°)

Parameters		Group A (n = 30)	Group B (n = 30)	Group C (n = 30)*
Stroke volume (mL)				
	Pretest	72 (62 - 82)	75 (59 - 86)	74 (61 - 97)
	Posttest	64 (54 - 76)†	72 (55 - 84)	79 (62 - 91)
Heart rate (bpm)				
	Pretest	71 (62 - 77)	73 (63 - 90)	79 (70 - 89)
	Posttest	75 (63 - 80)‡	73 (65 - 93)	81 (74 - 96)‡
Cardiac index (L/min/m^2^)				
	Pretest	2.85 (2.15 - 3.12)	3.10 (2.40 - 3.50)	3.21 (2.90 - 3.97)
	Posttest	2.50 (2.07 - 3.00)‡	3.00 (2.50 - 3.60)	3.61 (2.84 - 4.20)
Mean arterial pressure (mmHg)				
	Pretest	89 (82 - 95)	85 (80 - 94)	90 (84 - 100)
	Posttest	92 (89 - 100)§	88 (81 - 94)‡	91 (83 - 100)
Perfusion index (a.u.)				
	Pretest	4.84 (2.58 - 7.26)	3.73 (1.04 - 7.08)	4.38 (2.40 - 7.77)
	Posttest	3.11 (1.18 - 4.37)§	3.60 (1.03 - 5.91)	4.61(2.45 - 6.54)

* Data calculated with the first 30 septic patients;

† p < 0.01;

‡ p < 0.05;

§ p < 0.001 *versus* pretest values.

**Figure 3:**
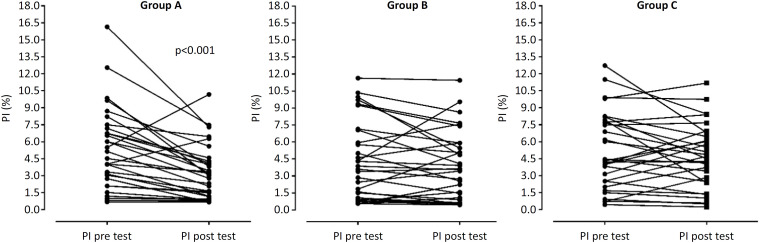
Individual values of the peripheral perfusion index pretest (0°) and posttest (semiorthostasis 60°).

Figure 3 shows the perfusion response after the semiorthostatic test for each individual in Groups A, B and C. Healthy patients mostly presented a significant decrease in the PI, whereas critical patients (septic and nonseptic) presented an unpredictable and heterogeneous response.

For this calculation, an n of 30 patients was used for each group. The first 30 patients were included in the group of septic patients (Group C). PI - perfusion index.

**Figure 4:**
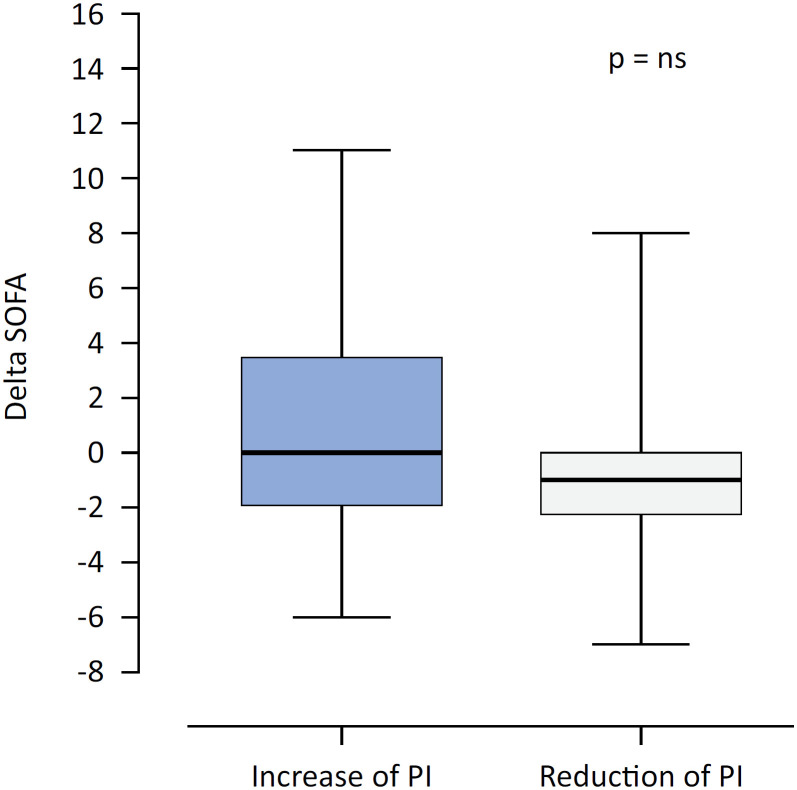
Perfusion index response after semiorthostasis as a predictor of organ dysfunction (delta sofa at 72 hours) in patients with sepsis.

The 28-day in-hospital mortality of patients with sepsis was 27% (25/92). Table 2 shows that the nonsurviving patients were older and had higher SOFA, APACHE II and procalcitonin scores. In addition, they presented higher CI and EF values, indicating a hyperkinetic state. As shown in figure 4, there was no statistically significant difference in the delta-SOFA score between patients with increased PI and those with reduced PI after the semiorthostatic test. However, as shown in table 3, there was a statistically significant decrease in the SOFA score within 72 hours only in the group with a reduced PI after the test compared with the group with an increased PI after the test.

SOFA - Sequential Organ Failure Assessment; PI - perfusion index; ns - not significant.

**Table 3:** Organ dysfunctions in septic patients with increased peripheral perfusion and decreased peripheral perfusion after the semiorthostatic stress test

Septic patients (Group C)	SOFA score		
	24 hours	72 hours
Increase perfusion index (n = 38)	5 (4 - 8)	6.25 (4 - 9.75)
Decrease in perfusion index (n = 54)	5 (4 - 6)	3 (2 - 6)*§

* p < 0.05 *versus* the "increase in the perfusion index group" at 72 hours (after adjustment via the Holm–Bonferroni method); † p < 0.05 *versus* the "increase in the perfusion index group" at 24 hours (after adjustment via the Holm–Bonferroni method).

## DISCUSSION

Alterations in the ANS response are fundamental components of the origin and development of organ dysfunction in sepsis.^(3,16)^ However, bedside ANS monitoring remains a challenge and is limited due to the scarcity of available methods.^(16)^ Clinical tools that can be used to characterize ANS dysfunction include heart rate variability (HRV), blood pressure variability, and baroreflex sensitivity.^(4)^ However, all these methods have not yet been disseminated in everyday clinical practice. The results of the present study, which used a simple test based on concepts of macro- and microcirculatory physiology, may contribute to increasing our knowledge of the role and importance of the ANS in sepsis.

The understanding of a normal and integrated hemodynamic response after postural changes is well supported by robust evidence.^(5,14,15)^ When the head is raised between 55° and 70°, approximately 10% of the blood volume (7 - 8mL/kg) is mobilized to the lower body, resulting in a decrease in SV. The effective compensatory response involves preserved neuroautonomic reflexes (particularly through arterial baroreceptors) and adequate venous/arterial reactivity, resulting in increases in HR, MAP and systemic peripheral resistance.^(5,15)^ The chosen angle in the present study (60°) promoted similar responses in healthy individuals, corroborating previous published findings.

In contrast to classic studies in the field, which used a macrohemodynamic assessment of these compensatory effects, the present study is the first to demonstrate that microcirculation changes occur concomitantly with macrocirculatory changes in humans in response to changes in posture and, more importantly, can be easily detected by a simple oximeter. In the context of ICU bedside tools and sepsis, this method is easy and prompt, can be repeated, and thus provides a powerful method for understanding the role of the ANS in sepsis.

Our results also indicate that a greater median effect was observed in the PI (- 35%) than in the MAP (+3%), HR (+5%), SV (-11%) and CI (-12%), suggesting that peripheral perfusion may be more sensitive to the evaluation of postural responses than other commonly used parameters.

Recent evidence clearly demonstrates a dissociation between the macro- and microcirculation in sepsis, as in the first hours of diagnosis, peripheral microcirculatory blood flow is more coupled with macrohemodynamic variations.^(17)^ However, in the latter hours and subsequent days, there is a loss of this hemodynamic coherence, and peripheral perfusion becomes an independent predictor of outcomes.^(2,17)^ In the present study, all septic patients were evaluated in the late phase of sepsis after resuscitation with fluids and achieving macrohemodynamic stability. Using this approach, a clear loss of the integrated sympathetic response to the test was demonstrated, both in peripheral perfusion and in macrohemodynamics, with only HR and MAP isolated responses persisting in the septic and critically ill groups, respectively. Although there was no difference between the medians of the groups in the PI assessment, there was interindividual variation, demonstrating important heterogeneity in the perfusion response of critically ill patients to semiorthostasis. Therefore, we hypothesized that in septic patients, a PI closer to normal could have a lower risk of worsening organic dysfunction.

The SNS is known to participate in neuro-mediated coupling between organs and blood flow.^(3,15)^ Additionally, its participation in immune regulation and thrombogenesis has been well reported in the literature.^(3,15)^ In patients with sepsis, there is a release of several inflammatory mediators that are linked to dysregulation of the appropriate autonomic response, disturbances in vasoreactivity, cardiac depression, immunosuppression and thrombogenesis, thus contributing to multiple organ dysfunctions.^(3,15)^ Our results revealed no statistically significant individual differences in delta-SOFA scores between patients with increased PI (abnormal sympathetic response) and those with reduced PI (normal sympathetic response). However, in the comparative analysis among the groups, there was an improvement in organic functions after 72 hours, as measured with the SOFA score, only in the group that had a reduced PI. A possible explanation for this apparent inconsistency may be attributed to the relatively low severity of the patient conditions. Furthermore, the first 30 septic patients included in the pilot study were younger and had a greater proportion of pulmonary sepsis (93 *versus* 48%) than did the final sample (Table 1S - Supplementary Material). Thus, despite the rigorous planning of the study, the factors mentioned may have influenced the evolution of organic dysfunctions, generating an underestimation of the sample for the desired effect size. Therefore, the prognostic potential of the test, especially in more severely ill patients, deserves future investigation.

A possible alternative interpretation of the results would be that although we suggest the PI test as a bedside autonomic assessment test, the perfusion response to semiorthostatic stress may be related to other pathophysiological pathways in sepsis. For example, vasoplegia (poor vascular response to vasoconstrictors, especially catecholamines) is a classic feature of sepsis, and it is commonly related to intense nitroso-oxidative stress secondary to the inflammatory cascade.^(18)^ Our results suggest that those patients who have shown increased PIs may be those with greater vasoplegia. This assumption is supported by the increased HR in septic patients, suggesting that the neural reflex pathway may be preserved and that the results may be due to poor vascular reactivity. However, as the critically ill group without sepsis also lost this integrated response, this hypothesis becomes less likely.

This test can also theoretically assess the unstressed blood volume in the venous capacitance system.^(19)^ In fact, microcirculation is considered a low-pressure system, and capillary perfusion pressure, a determinant of blood flow, may be more related to venous pressure than to arterial pressure.^(19,20)^ Therefore, patients with increased PI (which reduces venous return) may have high venous pressure, which impairs the perfusion gradient and prognosis. These possibilities are worthy of verification in future research.

The present study has several limitations. First, there was no direct measurement of parameters related to venous return (an important factor related to the test), such as central venous pressure. Additionally, the volume of fluids administered during the resuscitation phase was not measured. However, the exclusion of ventilated patients and the moment of assessment after resuscitation (less dependence on flow in relation to peripheral perfusion) were chosen to minimize these issues. Second, there was no comparison of this test with other autonomic assessment tests, such as HRV. Third, this test was performed only once and then again after fluid resuscitation. Since sepsis is a dynamic syndrome, these results cannot be extrapolated to the first hours of management or subsequent days. A future serial timepoint study is needed to rectify this issue. Fourth, it is worth mentioning that of the first 30 septic patients, 28 were diagnosed with COVID-19. This specific subgroup may not accurately represent the broader population of septic patients. Finally, a larger study with multivariate analysis and patients in more severe conditions, including subgroup analysis with COVID-19, is necessary to assess the prognostic value of the test clearly.

Despite these limitations, this study has considerable strengths in that it proposes a simple, inexpensive, easy-to-use and repeatable method to evaluate a field of study with few methods available. Therefore, new studies to address these limitations are feasible and inexpensive.

## CONCLUSION

The assessment of the peripheral perfusion response to semiorthostatic stress can differentiate the sympathetic-microvascular response between healthy, critically ill septic and nonseptic patients at the bedside. Furthermore, this test appears to have prognostic value. Therefore, this test may be useful in monitoring the autonomic nervous system and vascular reactivity of septic and critically ill patients.
