# Supplementary material for: Peripheral perfusion response to semiorthostatic stress: a simple method for assessing autonomic dysfunction in sepsis?
Source: Crit Care Sci. 2024 Nov 14;36:e20240090en. doi: 10.62675/2965-2774.20240090-en (PMC11634235; doi:10.62675/2965-2774.20240090-en)
Supplement: Supplementary file 1 [file 2965-2774-ccsci-36-e20240090en-Suppl01.pdf]

Critical Care Science
https://doi.org/10.62675/2965-2774.20240090-en

SUPPLEMENTARY MATERIAL

Peripheral perfusion response to semiorthostatic stress: a simple method for assessing autonomic dysfunction in sepsis?

Lilian Barth Guimarães[1], César Maistro Guimarães[2], Hipólito Carraro Junior[1], Jamil Assreuy Filho[3], Igor Alexandre Côrtes de Menezes[1]

Table 1S - Demographic, clinical and hemodynamic data in first 30 septic patients (Group C - Pilot study)

Parameters	Group C<br>n = 30
Clinical data	
Age (years)	35.2 (15.6)
Gender	
Male	23 (76)
Female	7 (24)
Comorbidities	
Diabetes Melitus	5 (16.6)
Hypertension	8 (26.6)
Chronic kidney disease	0
Cardíac failure	3 (10)
Hepatic failure	0
Cerebrovascular disease	1 (3.3)
Chronic lung disease	6 (20)
Cancer	3 (10)
Immunosuppressed	2 (6.6)
Source of infection	
Pulmonary	28 (93.3)
Abdominal	0
Urinary	2 (6.6)
Others	1 (3.3)
Positive culture	30 (100)
Positive hemoculture	3 (10)
COVID-19 confirmed	28 (93.3)
Scores and biomarkers	
SOFA - 24 hours	4.5 (1.16)
SOFA - 72 hours	5.16 (3.27)
APACHE II	10 (3.95)
C-reactive protein (mg/dL)	11.9 (7.5)
Procalcitonin (ng/mL)	8/0.20 (0.11 - 0.3)

Continue...

This is an open access article under the CC BY license (https://creativecommons.org/licenses/by/4.0/).

...continuation

Post-resuscitation hemodynamics	
Main arterial pressure (mmHg)	90 (84 - 99)
Heart rate (bpm)	81.4 (17.7)
Capillary refill time (s)	2 (2 - 3)
Perfusion index (a.u.)	4.38 (2.5 - 7.7)
Velocity time integral (cm)	21 (18.4 - 26.5)
Cardiac Index (L/minute/m²)	3.2 (2.9 - 3.9)
Ejection fraction	62.5 (57 - 65)
Cardiac output (L/minute)	6.2 (5.2 - 7.2)
Stroke volume (mL)	74 (62.5 - 91.5)
Percentage shortening (%)	33 (30 - 35)
Left ventricular outflow tract (cm)	1.9 (1.7 - 2)
Vasoactivedrugs use	0 (0)
Norepinephrine dose (μg/kg/minute)	0(0)
Vasopressin use, No. (%)	0 (0)
Arterial lactate (mmol/L)	24/1.5 (1.4 - 1.9)
Urinary output (mL/kg/hour)	24/0.74 (0.5 - 0.8)

SOFA - Sequential Organ Failure Assessment; APACHE II - Acute Physiology Chronic Health Evaluation II. Results expressed as mean (standard deviation). n (%), n/median (interquartile range) or median (interquartile range).
