# Supplementary material for: Peripheral perfusion response to semiorthostatic stress: a simple method for assessing autonomic dysfunction in sepsis?
Source: Crit Care Sci. 2024 Nov 14;36:e20240090en. doi: 10.62675/2965-2774.20240090-en (PMC11634235; doi:10.62675/2965-2774.20240090-en)
Supplement: Translation

# Translation: Resposta da perfusão periférica ao estresse semiortostático: um método simples para avaliar disfunção autonômica na sepse?

**DOI:** 10.62675/2965-2774.20240090-pt

**Authors:** Lilian Barth Guimarães, César Maistro Guimarães, Hipólito Carraro, Jamil Assreuy, Igor Alexandre Côrtes de Menezes

**Affiliations:** 1 Curitiba PR Brasil Unidade de Terapia Intensiva, Hospital de Clínicas, Universidade Federal do Paraná - Curitiba (PR), Brasil.; 2 São José dos Pinhais PR Brasil Unidade de Terapia Intensiva, Hospital e Maternidade São José dos Pinhais - São José dos Pinhais (PR), Brasil.; 3 Florianópolis SC Brasil Departamento de Farmacologia e Centro de Ciências Biológicas, Universidade Federal de Santa Catarina - Florianópolis (SC), Brasil.

**Keywords:** Sistema nervoso autônomo, Volume sistólico, Frequência cardíaca, Pressão arterial, Hemodinâmica, Microcirculação, Índice de perfusão, Prognóstico, Sepse

## Abstract

**Objetivo:**

Avaliar se a resposta do índice de perfusão ao estresse semiortostático pode ser usada para monitorar a resposta simpático-vascular em pacientes com sepse.

**Métodos:**

Três grupos foram estudados: Grupo A (30 pacientes saudáveis), Grupo B (30 pacientes em estado crítico sem sepse) e Grupo C (92 pacientes com sepse). Os pacientes foram submetidos a um teste de estresse semiortostático (elevação da cabeça de zero a 60°) e avaliaram-se a hemodinâmica, os valores do índice de perfusão e os dados de ultrassom cardíaco. Os valores do SOFA também foram avaliados em pacientes com sepse, comparando aqueles com índices de perfusão maiores e menores após o teste.

**Resultados:**

Após o teste, o Grupo A apresentou diminuição significativa no volume sistólico (p < 0,01) e no índice cardíaco (p < 0,05), com aumento da frequência cardíaca (p < 0,05) e da pressão arterial média (p < 0,001). Essas respostas não foram observadas nos Grupos B e C. Na análise individual do Grupo A, houve diminuição do índice de perfusão (p < 0,001), enquanto nos Grupos B e C a resposta foi heterogênea. Além disso, os pacientes com sepse com índice de perfusão menor após o teste tiveram uma queda significativa no escore SOFA em 72 horas em comparação com o grupo com índice de perfusão maior (p < 0,05). Entretanto, o escore delta-SOFA não diferiu nos grupos.

**Conclusão:**

A resposta do índice de perfusão ao estresse semiortostático em pacientes com sepse é um método simples e econômico na detecção da resposta simpático-microvascular à beira do leito e parece ter valor prognóstico.

## INTRODUÇÃO

A sepse é um fenômeno que continua a causar alta mortalidade.^(1)^ Verifica-se uma necessidade contínua de desenvolver novas abordagens clínicas e de monitoramento hemodinâmico para melhorar o prognóstico do paciente. Avanços significativos foram feitos, especialmente por meio da avaliação de distúrbios microvasculares à beira do leito com dispositivos portáteis.^(2)^ Quando esses dispositivos são usados, observa-se uma associação inequívoca entre esses distúrbios e desfechos desfavoráveis, e esses achados fornecem informações significativas acerca da fisiopatologia da sepse. Independentemente de os pacientes terem sido avaliados precoce ou tardiamente em relação à duração do tratamento hemodinâmico da sepse, constatou-se uma associação com prognóstico desfavorável.^(2)^

O sistema nervoso autônomo (SNA) e, especificamente, o sistema nervoso simpático (SNS) são cruciais para a regulação da perfusão. No entanto, em casos de sepse, há distúrbios na modulação do SNA, com os prováveis efeitos nocivos de mediadores simpáticos exógenos (por exemplo: drogas vasoativas) que podem contribuir para a regulação da perfusão.^(3)^ Muitas pesquisas relacionaram o comprometimento do SNS com a progressão das disfunções circulatórias, usando medidas diretas (intervenções cirúrgicas ou farmacológicas em animais) ou estimativas indiretas da atividade autonômica em pacientes/modelos animais.^(4)^ Além disso, a resposta do SNA pode ocorrer rapidamente para restaurar a homeostase. Os sinais clínicos de desregulação do SNA podem, assim, ser sinais de alerta precoce de um prognóstico desfavorável atribuído ao declínio cardiovascular.^(4)^

Além de seu papel na microcirculação, o SNA também é de importância central na homeostase ortostática, envolvendo respostas circulatórias e neuroendócrinas para manter a perfusão adequada dos órgãos, especialmente na postura ereta.^(5)^

Os oxímetros modernos calculam o índice de perfusão (IP), derivado de sinais fotopletismográficos. O IP é considerado uma medida não invasiva da perfusão microcirculatória periférica^(2,6)^ e tem alta sensibilidade a mudanças na estimulação simpática^(6)^ e a diferentes posições corporais.^(7)^ Recentemente, as respostas hemodinâmicas dos valores do IP a mudanças na posição corporal foram usadas como ferramenta de monitoramento na fluidoterapia, como visto no teste de elevação passiva das pernas.^(8)^ No entanto, o uso de variações no posicionamento como método para avaliar a resposta do SNA não foi testado.

Considerando o papel prognóstico dos distúrbios da microcirculação/perfusão e da disfunção autonômica na sepse, o papel do SNS na resposta circulatória às mudanças no posicionamento e a alta sensibilidade do IP às mudanças no posicionamento, nossa hipótese no presente estudo é que a resposta do IP à semiortostase pode constituir um teste simples e fácil à beira do leito para monitorar a atividade autonômica na sepse. O objetivo deste estudo foi avaliar se a resposta do IP ao estresse semiortostático pode ser usada para monitorar a resposta simpático-vascular em pacientes com sepse.

## MÉTODOS

### Desenho do estudo, local e participantes

A pesquisa foi aprovada pelo Comitê de Ética em Pesquisa em Seres Humanos do Complexo do Hospital de Clínicas da Universidade Federal do Paraná (UFPR), CAAE: 38786320.9.0000.0096, protocolo: 5.075.251, e todos os participantes ou responsáveis legais receberam e assinaram o Termo de Consentimento Livre e Esclarecido por escrito, consentindo em participar do estudo. Trata-se de um estudo observacional realizado em três unidades de terapia intensiva (UTIs) em Curitiba e São José dos Pinhais, no Paraná, entre fevereiro de 2020 e junho de 2023. Durante o estudo, foram considerados para inscrição todos os pacientes adultos consecutivos (≥ 18 anos) admitidos na UTI ou dentro de 24 horas após o início da sepse no caso de pacientes previamente admitidos por outras causas.

Os critérios de exclusão foram gravidez, morte dentro de 72 horas, uso de ventilação mecânica, arteriopatia obstrutiva grave, contraindicação clínica para mobilização no leito e recusa do paciente em participar do estudo.

De acordo com o consenso mais recente, identifica-se "sepse" pela presença de uma infecção associada à disfunção aguda de órgãos, conforme indicado por um aumento de dois pontos ou mais no *Sequential Organ Failure Assessment* (SOFA).^(9)^ O choque séptico é um subconjunto da sepse no qual, apesar da ressuscitação adequada com fluidos, a hiperlactatemia persistente (lactato arterial ≥ 2mmol/L) está associada à necessidade de vasopressores para atingir uma pressão arterial média (PAM) ≥ 65mmHg.^(9)^

### Protocolo do estudo

Todos os pacientes foram tratados seguindo um protocolo padrão adaptado das diretrizes da *Surviving Sepsis Campaign* (SSC).^(1)^ Os pacientes foram submetidos a fontes infecciosas, exame clínico, testes laboratoriais, coleta de cultura e aquisição de imagens. Na primeira hora, foram administrados antibióticos de amplo espectro conforme a fonte suspeita. Os pacientes receberam ressuscitação de fluidos com até 30mL/kg de fluido cristaloide durante as primeiras 3 horas do diagnóstico. Além disso, a ressuscitação com fluidos foi mantida, a critério do médico responsável e se houvesse uma indicação clínica individual, até que houvesse ausência de reação ao teste passivo de perna ou nenhuma variação respiratória no diâmetro da veia cava inferior. Se a PAM permanecesse abaixo de 65mmHg, doses progressivas de norepinefrina eram usadas para normalizar a PAM. A vasopressina foi associada à norepinefrina em casos refratários.

Os pacientes foram avaliados após a ressuscitação com fluidos entre 6 e 24 horas após o diagnóstico. Todos os pacientes foram avaliados após pelo menos 1 hora de estabilidade hemodinâmica (sem qualquer modificação das doses de drogas vasoativas, se em uso). As informações coletadas no momento da inscrição incluíram características demográficas, dados sobre sepse, resultados microbiológicos, doenças crônicas comórbidas e pontuações de gravidade, como as pontuações *Acute Physiology Chronic Health Evaluation II* (APACHE II) e SOFA. Além disso, foram obtidos parâmetros hemodinâmicos (PAM, frequência cardíaca [FC], débito urinário), gasométricos (saturação venosa central de oxigênio [ScvO_2_], diferença arteriovenosa de dióxido de carbono [Pv-aCO_2_]), biomarcadores (procalcitonina, proteína C-reativa [PCR]) e níveis de lactato antes da realização do teste semiortostático.

O IP representa a relação entre os componentes pulsáteis e não pulsáteis do sinal fotoelétrico proveniente da oximetria de pulso (Figura 1). O princípio da oximetria baseia-se em duas fontes de luz com diferentes comprimentos de onda (660nm e 940nm) emitidas através do leito vascular cutâneo de um dedo ou lóbulo da orelha. Usando um sistema de dois comprimentos de onda, o componente não pulsátil (como tecido conjuntivo, osso e sangue venoso) é descartado, e o componente pulsátil é usado para calcular a saturação arterial de oxigênio. Como somente o componente pulsátil do sinal varia em função das flutuações do fluxo sanguíneo (secundárias à vasodilatação ou vasoconstrição), o IP é considerado um método válido para avaliar a perfusão periférica.^(10)^ O IP foi medido colocando-se uma sonda de oxímetro (Mindray, Shenzen, China) no dedo indicador.

**Figura 1:** Índices de perfusão periférica e componentes pulsáteis e não pulsáteis.

Os intensivistas não tinham conhecimento dos valores do teste para evitar possíveis vieses de tratamento. Por fim, os pacientes foram seguidos por 28 dias após o diagnóstico ou até a alta.

### Ultrassom cardíaco

Todos os ecocardiogramas bidemensionais transtorácicos foram realizados pelo mesmo médico, que não tinha conhecimento dos dados clínicos e era devidamente treinado em ecocardiografia. Utilizou-se um aparelho de ultrassom da Philips (Affiniti 70G; Andover, MA, Estados Unidos). Neste estudo, os parâmetros coletados incluíram a fração de ejeção (FE), a via de saída do ventrículo esquerdo, o volume sistólico (VS) e a integral velocidade-tempo (IVT). O índice cardíaco (IC) e o débito cardíaco (DC) foram calculados a partir dos dados.

### O teste semiortostático

Os pacientes foram mantidos em posição de decúbito dorsal, com a cabeceira da cama a zero grau por 5 minutos. O IP, o tempo de enchimento capilar (TEC) e os dados de ultrassom cardíaco (VS, IC, DC, FE) foram registrados posteriormente. Após esse período, a cabeceira da cama foi elevada a 60° e mantida nessa posição por 3 minutos, e todos os dados foram registrados. Esse protocolo experimental baseou-se na avaliação padronizada da hipotensão ortostática em pacientes ambulatoriais.^(11)^

Os participantes foram posteriormente divididos em três grupos:

- Grupo A - grupo controle saudável: funcionários da UTI sem evidência de infecções agudas.
- Grupo B - pacientes em estado crítico sem sepse: pacientes internados na UTI por outras doenças sem atender ao critério de diagnóstico de sepse.
- Grupo C - pacientes com sepse (< 24 horas): pacientes admitidos na UTI e diagnosticados com sepse.

Além disso, os pacientes com sepse foram divididos em dois grupos, a saber, aqueles com perfusão periférica aumentada após o teste (IP pós-teste - IP pré-teste > zero unidades arbitrárias) e pacientes com perfusão periférica diminuída após o teste (IP pós-teste - IP pré-teste ≤ zero unidades arbitrárias).

### Desfechos

O desfecho primário deste estudo foi a comparação das variáveis hemodinâmicas e dos IPs após o teste semiortostático entre os Grupos A, B e C.

O desfecho secundário deste estudo foi a comparação das disfunções orgânicas nos grupos em que o IP aumentou ou diminuiu após o teste, conforme medido pelos escores SOFA em série (24 e 72 horas) e pelo escore delta-SOFA após 72 horas.

### Análise estatística

O teste de Shapiro-Wilk foi usado para avaliar a normalidade da amostra. Os dados paramétricos são apresentados como média ± desvio-padrão (DP); os dados não paramétricos são apresentados como mediana e intervalo interquartil. As proporções são representadas como porcentagens. As comparações entre os grupos em termos de escore delta-SOFA e hemodinâmica foram realizadas por meio do teste de Mann-Whitney. Para a avaliação em série do escore SOFA (24 e 72 horas), foram usados o teste de Mann-Whitney (intergrupo) e o teste dos postos sinalizados de Wilcoxon (intragrupo) com correção para comparações múltiplas por meio do método de Holm-Bonferroni. No cálculo do tamanho da amostra, usamos dados de um estudo-piloto com a inclusão de 30 pacientes com sepse na primeira amostra (que foi usada para comparação com os Grupos B e C). Assim, os pacientes cujo IP diminuiu constituíram dois terços da amostra, e esse grupo teve um escore delta-SOFA médio (72 horas) de zero pontos, com DP de aproximadamente três pontos. Além disso, neste estudo, uma diferença mínima de dois pontos entre os grupos foi necessária para a relevância clínica em relação ao escore delta-SOFA.^(12)^ Como os dados são não paramétricos, a amostra também foi inflada em 15%.^(13)^ Considerando todos esses aspectos, estimamos amostra total de 92 pacientes com sepse (Grupo C).

Para a análise clínico-hemodinâmica, os tamanhos das amostras dos Grupos A e B foram calculados a partir de estudos anteriores na literatura.^(14,15)^ Como resultado, estimamos 30 pacientes em cada grupo para verificar uma diminuição de aproximadamente 25% na VS após o teste. O poder amostral escolhido foi de 80%, com um erro alfa de 5%.

Este estudo seguiu as diretrizes do STROBE para relatar os resultados.

## RESULTADOS

Um total de 152 pacientes foi incluído no estudo do teste semiortostático, conforme mostrado na figura 2. É importante ressaltar que grande parte deste estudo foi realizada durante a pandemia da doença causada pelo coronavírus 2019 (COVID-19) e, devido às limitações internas do serviço em decorrência das dificuldades conhecidas de assistência médica durante esse período, a viabilidade da coleta de dados foi frequentemente interrompida, atrasando a conclusão do estudo. Os dados clínicos, demográficos e hemodinâmicos são apresentados na tabela 1. Esses dados descrevem caracteristicamente uma população heterogênea e em estado crítico. Os pacientes em estado crítico tinham alta prevalência de hipertensão e diabetes, além de comorbidades comuns em outras coortes de terapia intensiva (como câncer e doença pulmonar crônica). Além disso, os pacientes selecionados incluíam pacientes cirúrgicos e não cirúrgicos (nos grupos com sepse e sem sepse).

**Figura 2:** Pacientes do estudo.

UTI - unidade de terapia intensiva.

**Tabela 1:** Dados demográficos, clínicos e hemodinâmicos em controles saudáveis (Grupo A), pacientes críticos sem sepse (Grupo B) e pacientes com sepse (Grupo C)

Parâmetros			Grupo A n = 30	Grupo B n = 30	Grupo C n = 92	Grupo C Sobreviventes n = 67	Grupo C Não sobreviventes n = 25	Valor de p
Dados clínicos								
	Idade (anos)		37,3 (11)	62,3 (16,17)	55,2 (16,6)	52,9 (16,6)	61,9 (11,45)	0,007*
	Sexo							
		Masculino	18 (60)	11 (36,6)	60 (65,2)	42 (62,6)	18 (72)	0,46
		Feminino	12 (40)	19 (66,3)	32 (34,8)	25 (37,3)	7 (28)	
Comorbidades								
	*Diabetes mellitus*		0	10 (33,3)	23 (25)	17 (25,4)	6 (24)	> 0,99
	Hipertensão		0	16 (53,3)	38 (41)	27 (40,3)	11 (44)	0,81
	Doença renal crônica		0	3 (10)	7 (7,6)	3 (4,5)	4 (16)	0,84
	Insuficiência cardíaca		0	9 (30)	17 (18,5)	14 (20,9)	3 (12)	0,38
	Insuficiência hepática		0	1(3,3)	7 (7,6)	4 (6)	3 (12)	0,38
	Doença cerebrovascular		0	3 (10)	4 (4,3)	3 (4,5)	1 (4)	> 0,99
	Doença pulmonar crônica		0	5 (16,6)	15 (16,3)	11 (16,4)	4 (16)	> 0,99
	Câncer		0	0 (0)	22 (23,9)	13 (19,4)	9 (36)	0,10
	Imunossupressão		0	1 (3,3)	15 (16,3)	12 (17,9)	3 (12)	0,75
Fonte de infecção								
	Pulmonar		N/A	-	45 (48,9)	31 (46,3)	14 (56)	0,48
	Abdominal		N/A	-	22 (24)	15 (22,4)	7 (28)	0,59
	Urinária		N/A	-	13 (14,1)	13 (19,4)	0 (0)	0,01*
	Outra		N/A	-	12 (13)	8 (11,9)	4 (16)	0,79
	Cultura positiva		N/A	-	72 (78)	52 (77,6)	20 (80)	> 0,99
	Hemocultura positiva		N/A	-	13 (14,1)	14 (20,9)	5 (20)	> 0,99
	COVID-19 confirmada		N/A	-	32 (34,8)	21 (31,3)	11 (44)	0,32
Escores e biomarcadores								
	SOFA 24 horas		N/A	2,2 (1,9)	5,9 (2,7)	5,5 (2,5)	6,7 (3,1)	> 0,99
	SOFA 72 horas		N/A	1,6 (1,6)	5,8 (3,9)	4,5 (3,0)	8,9 (4)	0,0001†
	APACHE II		N/A	10,7 (5,6)	13,7 (5,6)	12,8 (5,2)	16 (5,9)	0,004‡
	Proteína C-reativa (mg/dL)		N/A	4,4 (6,8)	17,2 (11,2)	17,2 (11,2)	17,6 (11,2)	0,71
	Procalcitonina (ng/mL)		N/A	-	12/0,2 (0,1 - 0,3)	10/0,15 (0,08 - 0,3)	3/0,3 (0,2 - 3,3)	0,001‡
Hemodinâmica pós-ressuscitação								
	Pressão arterial média (mmHg)		89 (82 - 93,5)	85 (80,2 - 93)	85 (76 - 92)	85 (77 - 94)	84 (74 - 90)	0,29
	Frequência cardíaca (bpm)		70,6 (11,8)	76 (12)	94 (22)	95 (20)	93 (26)	0,42
	Tempo de reenchimento capilar (s)		2 (2 - 2)	2 (2 - 3)	3 (2 - 3)	2 (2 - 3)	3 (2 - 3)	0,28
	Índice de perfusão (u.a.)		5,3 (2,8 - 7,4)	3,7 (1,2 - 6,8)	3,2 (1,4 - 6,0)	3,2 (1,4 - 5,9)	3,1 (0,9 - 6,2)	0,33
	Integral velocidade-tempo (cm)		22,5 (2 - 24)	25,5 (19 - 27,2)	19,8 (17 - 23,5)§	19,3 (17,7 - 22)	22 (18,7 - 28,6)	0,41
	Índice cardíaco (L/minuto/m²)		2,8 (2,2 - 3,1)	3,1 (2,4 - 3,5)	3,2 (2,8 - 3,9)§	3,1 (2,2 - 3,5)	3,7 (3,3 - 4,2)	0,021*
	Fração de ejeção		66 (65 - 68)	55 (46,5 - 61,5)	61 (56,8 - 65)§	63,6 (58 - 67)	56,5 (55 - 62,2)	0,02*
	Débito cardíaco (L/minuto)		5,1 (4 - 5,9)	5 (4,5 - 6,6)	6,2 (5,1 - 7,1)§	5,7 (4,6 - 7)	6,7 (6 - 7,5)	0,14
	Volume sistólico (mL)		72,5 (62 - 80,7)	75 (59 - 86)	74 (61 - 90,5)§	73 (58,7 - 87)	80 (71 - 102)	0,25
	Encurtamento (%)		36 (35 - 38)	30 (23,2 - 32)	33 (30 - 35)§	34 (32 - 36)	29,6 (29 - 31,5)	0,03*
	Via de saída do ventrículo esquerdo (cm)		2,0 (1,9 - 2,1)	2 (1,9 - 2,1)	2,1 (2,1 - 2,2)§	2,1 (2,1 - 2,12)	2,2 (2,1 - 2,2)	0,09
	Uso de drogas vasoativas		N/A	0 (0)	28 (30,4)	20 (29,8)	8 (32)	> 0,99
	Dose de norepinefrina (µg/kg/minuto)		N/A	0 (0)	0,3 (0,1 - 0,65)	0,25 (0,1 - 0,6)	0,5 (0,2 - 0,9)	0,69
	Uso de vasopressina		N/A	1 (1,63)	4 (4,3)	1 (1,5)	3 (12)	0,05
	Lactato arterial (mmol/L)		N/A	18/1,2 (0,8 - 1,7)	71/1,8 (1,5 - 2,7)	50/1,8 (1,5 - 2,6)	21/1,6 (1,3 - 2,9)	0,62
	Débito urinário (mL/kg/hora)		N/A	26/0,7 (0,7 - 0,8)	62/0,6 (0,5 - 0,8)	48/0,65 (0,49 - 0,8)	14/0,5 (0,5 - 0,7)	0,09

SOFA - *Sequential Organ Failure Assessment*; APACHE II - *Acute Physiology Chronic Health Evaluation II*.

* = p < 0,05;

† = p < 0,001;

‡ = p < 0,01;

§ os dados foram calculados a partir dos primeiros 32 pacientes com sepse. Valor de p: sobreviventes do Grupo C *versus* não sobreviventes. Resultados expressos como médias (desvios-padrão), n (%), n/mediana (intervalo interquartil) ou mediana (intervalo interquartil).

Conforme mostrado na tabela 2, o teste de estresse semiortostático causou uma redução do VS e do IC no grupo controle saudável (Grupo A), com aumento compensatórios da FC e da PAM. Concomitantemente a essas respostas macro-hemodinâmicas, houve diminuição significativa na perfusão periférica medida pelo IP. Essa resposta integrada foi perdida nos pacientes críticos sem sepse (Grupo B) e com sepse (Grupo C).

**Tabela 2:** Macro-hemodinâmica e perfusão periférica em controles saudáveis (Grupo A), pacientes críticos sem sepse (Grupo B) e pacientes com sepse (Grupo C) antes (0°) e após o teste de estresse semiortostático (60°)

Parâmetros		Grupo A (n = 30)	Grupo B (n = 30)	Grupo C (n = 30)*
Volume sistólico (mL)				
	Pré-teste	72 (62 - 82)	75 (59 - 86)	74 (61 - 97)
	Pós-teste	64 (54 - 76)†	72 (55 - 84)	79 (62 - 91)
Frequência cardíaca (bpm)				
	Pré-teste	71 (62 - 77)	73 (63 - 90)	79 (70 - 89)
	Pós-teste	75 (63 - 80)‡	73 (65 - 93)	81 (74 - 96)‡
Índice cardíaco (L/min/m^2^)				
	Pré-teste	2,85 (2,15 - 3,12)	3,10 (2,40 - 3,50)	3,21 (2,90 - 3,97)
	Pós-teste	2,50 (2,07 - 3,00)‡	3,00 (2,50 - 3,60)	3,61 (2,84 - 4,20)
Pressão arterial média (mmHg)				
	Pré-teste	89 (82 - 95)	85 (80 - 94)	90 (84 - 100)
	Pós-teste	92 (89 - 100)§	88 (81 - 94)‡	91 (83 - 100)
Índice de perfusão (u.a.)				
	Pré-teste	4,84 (2,58 - 7,26)	3,73 (1,04 - 7,08)	4,38 (2,40 - 7,77)
	Pós-teste	3,11 (1,18 - 4,37)§	3,60 (1,03 - 5,91)	4,61(2,45 - 6,54)

* Dados calculados com os primeiros 30 pacientes com sepse;

† p < 0,01;

‡ p < 0,05;

§ p< 0,001 *versus* valores do pré-teste.

A figura 3 mostra a resposta de perfusão após o teste semiortostático em cada indivíduo dos Grupos A, B e C. Os pacientes saudáveis apresentaram, em sua maioria, uma diminuição significativa no IP, enquanto os críticos (com sepse e sem sepse) apresentaram uma resposta imprevisível e heterogênea.

**Figura 3:** Valores individuais do índice de perfusão periférica pré-teste (0°) e pós-teste (semiortostase a 60°).

Para esse cálculo, utilizou-se n de 30 pacientes por grupo. Os primeiros 30 pacientes foram incluídos no grupo de pacientes com sepse (Grupo C). IP - índice de perfusão.

A mortalidade hospitalar em 28 dias dos pacientes com sepse foi de 27% (25/92). A tabela 2 mostra que os pacientes que não sobreviveram eram mais velhos e tinham escores SOFA, APACHE II e procalcitonina mais altos. Além disso, eles apresentaram valores mais altos de IC e EF, indicando um estado hipercinético. Conforme mostrado na figura 4, não houve diferença estatisticamente significativa no escore delta-SOFA nos pacientes com IP aumentado e com IP diminuído após o teste semiortostático. No entanto, conforme mostrado na tabela 3, houve uma diminuição estatisticamente significativa no escore SOFA em 72 horas somente no grupo com IP diminuído após o teste em comparação com o grupo com IP aumentado após o teste.

**Figura 4:** Resposta do índice de perfusão após semiortostase como preditor de disfunção orgânica (delta-SOFA em 72 horas) em pacientes com sepse.

SOFA - *Sequential Organ Failure Assessment*; IP - índice de perfusão; ns - não significativo.

**Tabela 3:** Disfunções orgânicas em pacientes com sepse com perfusão periférica aumentada e perfusão periférica diminuída após o teste de estresse semiortostático

Pacientes com sepse (Grupo C)	SOFA	
	24 horas	72 horas
Aumento do índice de perfusão (n = 38)	5 (4 - 8)	6,25 (4 - 9,75)
Diminuição do índice de perfusão (n = 54)	5 (4 - 6)	3 (2 - 6)*†

* p < 0,05 *versus* o "grupo aumento do índice de perfusão" em 72 horas (após ajuste pelo método de Holm-Bonferroni); † p < 0,05 *versus* o "grupo aumento do índice de perfusão" em 24 horas (após ajuste pelo método de Holm-Bonferroni). SOFA - *Sequential Organ Failure Assessment*

## DISCUSSÃO

As alterações na resposta do SNA são componentes fundamentais da origem e do desenvolvimento da disfunção orgânica na sepse.^(3,16)^ No entanto, a monitoração do SNA à beira do leito continua sendo um desafio e é limitada devido à escassez de métodos disponíveis.^(16)^ As ferramentas clínicas que podem ser usadas para caracterizar a disfunção do SNA incluem a variabilidade da frequência cardíaca (VFC), a variabilidade da pressão arterial e a sensibilidade barorreflexa.^(4)^ No entanto, todos esses métodos ainda não foram disseminados na prática clínica cotidiana. Os resultados do presente estudo, que utilizou um teste simples embasado em conceitos de fisiologia macro e microcirculatória, podem contribuir para aumentar nosso conhecimento do papel e da importância do SNA na sepse.

A compreensão de uma resposta hemodinâmica normal e integrada após alterações posturais é bem apoiada por evidências robustas.^(5,14,15)^ Quando a cabeça é elevada entre 55° e 70°, aproximadamente 10% do volume de sangue (7 - 8mL/kg) é mobilizado para a parte inferior do corpo, resultando em uma diminuição do VS. A resposta compensatória efetiva envolve reflexos neuroautonômicos preservados (particularmente por meio de barorreceptores arteriais) e reatividade venosa/arterial adequada, resultando em aumento na FC, PAM e resistência periférica sistêmica.^(5,15)^ O ângulo escolhido no presente estudo (60°) promoveu respostas semelhantes em indivíduos saudáveis, corroborando achados publicados anteriormente.

Em contraste com estudos clássicos na área, que usaram uma avaliação macro-hemodinâmica desses efeitos compensatórios, o presente estudo é o primeiro a demonstrar que as alterações na microcirculação ocorrem concomitantemente com as alterações macrocirculatórias em humanos em resposta a alterações na postura e, mais importante, podem ser facilmente detectadas por um oxímetro simples. No contexto das ferramentas de beira de leito da UTI e da sepse, esse método é fácil e rápido, pode ser repetido e, portanto, oferece um método poderoso para compreender a função do SNA na sepse.

Nossos resultados também indicam que foi observado um efeito mediano maior no IP (- 35%) do que na PAM (+3%), FC (+5%), VS (-11%) e IC (-12%), sugerindo que a perfusão periférica pode ser mais sensível à avaliação das respostas posturais do que outros parâmetros comumente usados.

Evidências recentes demonstram claramente uma dissociação entre a macro e a microcirculação na sepse, já que, nas primeiras horas do diagnóstico, o fluxo sanguíneo da microcirculação periférica está mais associado às variações macro-hemodinâmicas.^(17)^ Entretanto, nas últimas horas e nos dias subsequentes, há uma perda dessa coerência hemodinâmica, e a perfusão periférica se torna um preditor independente de desfechos.^(2,17)^ No presente estudo, todos os pacientes com sepse foram avaliados na fase tardia da sepse, após a ressuscitação com fluidos e a obtenção da estabilidade macro-hemodinâmica. Ao usar essa abordagem, foi demonstrada clara perda da resposta simpática integrada ao teste, tanto na perfusão periférica quanto na macro-hemodinâmica, com apenas respostas isoladas de FC e PAM persistindo nos grupos com sepse e em estado crítico, respectivamente. Embora não tenha havido diferença entre as medianas dos grupos na avaliação do IP, houve variação interindividual, demonstrando importante heterogeneidade na resposta de perfusão de pacientes críticos à semiortostase. Portanto, levantamos a hipótese de que, em pacientes com sepse, um IP mais próximo do normal poderia ter um risco menor de agravamento da disfunção orgânica.

Sabe-se que o SNS participa do acoplamento neuromediado entre os órgãos e o fluxo sanguíneo.^(3,15)^ Além disso, sua participação na regulação imunológica e na trombogênese tem sido bem relatada na literatura.^(3,15)^ Em pacientes com sepse, há uma liberação de vários mediadores inflamatórios que estão ligados à desregulação da resposta autonômica adequada, aos distúrbios na vasorreatividade, à depressão cardíaca, à imunossupressão e à trombogênese, contribuindo para múltiplas disfunções orgânicas.^(3,15)^ Nossos resultados não revelaram diferenças individuais estatisticamente significativas nos escores delta-SOFA em pacientes com IP aumentado (resposta simpática anormal) e aqueles com IP diminuído (resposta simpática normal). No entanto, na análise comparativa dos grupos, houve melhora nas funções orgânicas após 72 horas apenas no grupo com IP reduzido, conforme medido com o escore SOFA. Uma possível explicação para essa aparente inconsistência pode ser atribuída à gravidade relativamente baixa das condições dos pacientes. Além disso, os primeiros 30 pacientes com sepse incluídos no estudo-piloto eram mais jovens e tinham uma proporção maior de sepse pulmonar (93% *versus* 48%) do que a amostra final (Tabela 1S - Material Suplementar). Assim, apesar do rigoroso planejamento do estudo, os fatores mencionados podem ter influenciado a evolução das disfunções orgânicas, gerando uma subestimação da amostra quanto ao tamanho do efeito desejado. Portanto, o potencial prognóstico do teste, especialmente em pacientes mais graves, merece investigação futura.

Uma possível interpretação alternativa dos resultados seria que, apesar de sugerirmos o teste do IP como de avaliação autonômica à beira do leito, a resposta de perfusão ao estresse semiortostático pode estar relacionada a outras vias fisiopatológicas na sepse. Por exemplo, a vasoplegia (resposta vascular deficiente a vasoconstritores, especialmente catecolaminas) é uma característica clássica da sepse e está comumente relacionada ao intenso estresse nitroso-oxidativo secundário à cascata inflamatória.^(18)^ Nossos resultados sugerem que os pacientes que apresentaram IPs aumentados podem ser aqueles com maior vasoplegia. Essa suposição é apoiada pelo aumento da FC em pacientes com sepse, sugerindo que a via reflexa neural pode estar preservada e que os resultados podem ser devidos à baixa reatividade vascular. Entretanto, como o grupo de pacientes graves sem sepse também perdeu essa resposta integrada, essa hipótese se torna menos provável.

Esse teste também pode, teoricamente, avaliar o volume de sangue não estressado no sistema de capacitância venosa.^(19)^ De fato, a microcirculação é considerada um sistema de baixa pressão, e a pressão de perfusão capilar, um determinante do fluxo sanguíneo, pode estar mais relacionada à pressão venosa do que à pressão arterial.^(19,20)^ Portanto, pacientes com IP aumentado (o qual reduz o retorno venoso) podem ter pressão venosa alta, o que prejudica o gradiente de perfusão e o prognóstico. Essas possibilidades merecem ser verificadas em pesquisas futuras.

O presente estudo tem várias limitações. Primeiro, não houve medição direta de parâmetros relacionados ao retorno venoso (um fator importante relacionado ao teste), como a pressão venosa central. Além disso, o volume de fluidos administrados durante a fase de ressuscitação não foi medido. Entretanto, a exclusão de pacientes ventilados e o momento da avaliação após a ressuscitação (menor dependência do fluxo em relação à perfusão periférica) foram escolhidos para minimizar esses problemas. Segundo, não houve comparação desse teste com outros testes de avaliação autonômica, como a VFC. Terceiro, esse teste foi realizado apenas uma vez e depois novamente após a ressuscitação com fluidos. Como a sepse é uma síndrome dinâmica, esses resultados não podem ser extrapolados nas primeiras horas de tratamento ou nos dias subsequentes. É necessário conduzir um futuro estudo em série cronológico para corrigir esse problema. Em quarto lugar, vale a pena mencionar que, dos primeiros 30 pacientes com sepse, 28 foram diagnosticados com COVID-19. Esse subgrupo específico pode não representar com precisão a população mais ampla de pacientes com sepse. Por fim, é necessário um estudo maior com análise multivariada e pacientes em condições mais graves, incluindo análise de subgrupo com COVID-19, para avaliar claramente o valor prognóstico do teste.

Apesar dessas limitações, este estudo tem pontos fortes consideráveis, pois propõe um método simples, econômico, fácil de usar e repetível para avaliar um campo de estudo com poucos métodos disponíveis. Portanto, novos estudos para abordar essas limitações são viáveis e econômicos.

## CONCLUSÃO

A avaliação da resposta de perfusão periférica ao estresse semiortostático pode diferenciar a resposta simpática-microvascular em pacientes com sepse e sem sepse, saudáveis e em estado crítico à beira do leito. Além disso, esse teste parece ter valor prognóstico. Portanto, esse teste pode ser útil para monitorar o sistema nervoso autônomo e a reatividade vascular de pacientes com sepse e em estado crítico.
